# Adrenal tumor microenvironment: hormone–immune crosstalk, molecular heterogeneity, and immunotherapeutic opportunities

**DOI:** 10.3389/fimmu.2026.1853359

**Published:** 2026-06-26

**Authors:** Shuang Chen, Xin Gao

**Affiliations:** 1 Department of Operating Room 1, the First Hospital of Jilin University, Changchun, Jilin, China; 2 Department of Urology, the First Hospital of Jilin University, Changchun, Jilin, China

**Keywords:** adrenal tumors, adrenocortical carcinoma, hormone–immune crosstalk, immune checkpoint blockade, pheochromocytoma/paraganglioma, tumor microenvironment

## Abstract

Adrenal tumors comprise a heterogeneous spectrum ranging from functional adenomas to aggressive adrenocortical carcinoma (ACC) and pheochromocytoma/paraganglioma (PHEO/PPGL) with metastatic potential. Although traditionally interpreted through hormone excess and oncogenic alterations, current evidence indicates that these tumors are endocrine-shaped immune ecosystems in which hormone secretion, molecular subtype, stromal architecture, metabolic stress, and immune infiltration interact to determine tumor behavior and therapeutic vulnerability. Across subtypes, distinct immune–stromal states emerge: aldosterone-producing adenoma (APA) contains M2-polarized macrophages, specialized endothelial subsets, and metabolic heterogeneity; cortisol-producing adenoma (CPA) is characterized by local glucocorticoid-driven immunosuppression and altered macrophage and T-cell states; ACC is relatively immune-depleted and shaped by glucocorticoid signaling, hypoxia, senescence, and myeloid suppression; and PHEO/PPGL exhibits subtype-dependent angiogenic and immune features linked to catecholamine biology and pseudohypoxia. The strongest human evidence supports cortisol-associated immune remodeling in CPA, macrophage-rich niches in APA, and immune ecotypes in ACC, whereas CAF-mediated immune exclusion, ion-channel-driven immune regulation, and several metabolite-based mechanisms remain largely extrapolative. Clinically, immune checkpoint blockade has shown modest and heterogeneous activity, especially in ACC, where PD-L1 and tumor mutational burden have not consistently predicted response, while selected PPGL subsets may be biologically more permissive. These findings support a model in which adrenal tumors should be classified not only by histology and hormone excess, but also by endocrine–immune microenvironmental states, with implications for biomarker development and rational combination therapies.

## 1 Introduction

### 1.1 Clinical and biological heterogeneity of adrenal tumors

The adrenal glands are composed of a mesoderm-derived cortex and a neural crest-derived medulla. The cortex is zonated to produce mineralocorticoids, glucocorticoids, and androgens, while the medulla synthesizes catecholamines. The transcription factor SF-1 (NR5A1) is a master regulator of adrenocortical development and a hallmark of adrenocortical tumors (1, 2). Adrenal aging remodels cortical architecture and immune surveillance, potentially creating a permissive niche for tumor initiation (3), and a capsular stem/progenitor cell compartment may serve as a cell-of-origin for cortical neoplasms (4, 5).

Adrenal tumors display striking clinical heterogeneity. Benign cortical adenomas include aldosterone-producing adenomas (APA), which cause primary aldosteronism (6), and cortisol-producing adenomas (CPA), which lead to Cushing’s syndrome (7). Although typically cured by surgery, both APA and CPA are associated with substantial cardiometabolic morbidity (6, 8). In contrast, adrenocortical carcinoma (ACC) is rare but highly aggressive, with a 5-year survival below 35% in advanced disease (9, 10). Medullary pheochromocytomas and paragangliomas (PPGL) are often indolent, yet a subset, particularly those with SDHB mutations, develops metastases (11). This diverse clinical spectrum—from hormonally active but benign lesions to rapidly lethal cancers—motivates the search for biological determinants of hormone secretion, recurrence, and malignant progression.

Notably, the adrenal gland is not a classical immune-privileged organ like the eye or testis (12, 13); T cells and other immune populations are detectable in adrenal tissues and tumors (7, 14, 15). However, local production of steroids and catecholamines, together with stromal and metabolic constraints, imposes a state of context-dependent immune restraint (12, 16). Adrenal immune regulation is therefore best conceptualized as endocrine-associated immune modulation rather than absolute privilege.

### 1.2 The adrenal tumor microenvironment: a hormone-shaped immune ecosystem

The tumor microenvironment (TME)—comprising immune cells, cancer-associated fibroblasts, endothelial cells, and extracellular matrix—is a central determinant of tumor progression, hormone secretion, and therapy response. In ACC, TME-based molecular classifications identify subtypes with distinct immune profiles and prognostic significance (17). Metabolic alterations, such as deregulated hyaluronan metabolism, drive immunosuppression by promoting PD-L1^+^ tumor-associated macrophages (18), and phosphoethanolamine accumulation impairs CD8^+^ T cell function (19). Even in benign adenomas, the TME is clinically relevant: immune and stromal components can modulate steroidogenic activity, contribute to heterogeneity in hormone excess, and help identify rare cases with recurrence or malignant potential.

What sets the adrenal TME apart from that of common solid tumors is the pervasive bidirectional crosstalk between hormone secretion and immune function. Cortisol-producing tumors generate a local immunosuppressive milieu that skews macrophage polarization and dampens T cell activity (7), while catecholamine secretion in PPGL can alter local metabolism and suppress anti-tumor immunity (18, 20). Conversely, microenvironment-derived factors such as hypoxia and cytokines may feedback on hormone synthesis. Despite these insights, the adrenal TME remains incompletely understood. Most studies are small and retrospective, and causal links between specific hormonal outputs and immune phenotypes have rarely been demonstrated directly in human adrenal tumors. Much of the current mechanistic understanding is extrapolated from other malignancies or preclinical models, underscoring a critical gap in adrenal-specific immunobiology.

### 1.3 Scope and evidence classification of this review

In this review, we conceptualize adrenal tumors as hormone-producing immune ecosystems. We aim to (i) detail the cellular and acellular components of the adrenal TME, (ii) examine how molecular heterogeneity, steroidogenesis, and catecholamine biology shape immune–stromal states across APA, CPA, ACC, and PPGL, and (iii) critically assess immunotherapeutic opportunities, including immune checkpoint blockade and TME-directed strategies. Throughout, we explicitly distinguish direct adrenal tumor evidence from preclinical findings and extrapolations. To avoid overinterpretation, we classify supporting evidence into four levels: (A) adrenal tumor-specific clinical/translational studies, (B) adrenal tumor-specific preclinical models, (C) endocrine-biological mechanisms not directly validated in adrenal tumors, and (D) extrapolations from other malignancies. Claims based on Level C or D evidence are presented as hypotheses.

## 2 The adrenal TME: cellular and acellular architecture

### 2.1 Cellular components: immune cells, fibroblasts, and endothelium

Single-nucleus and spatial transcriptomic studies of human adrenal tumors have identified diverse immune and stromal populations, including CD68^+^ and CD163^+^ tumor-associated macrophages (TAMs) that frequently display an M2-like polarization pattern, cancer-associated fibroblasts (CAFs), and abnormal tumor-associated endothelial cells (14, 15). T cell infiltration has been documented in both cortical and medullary tumors, though its density and functional orientation vary markedly with histology and molecular subtype (7, 21). By extrapolation from other malignancies, CAFs are thought to modulate T cell function and remodel the extracellular matrix, while tumor-associated endothelial cells may exhibit aberrant angiogenesis and contribute to immune exclusion (22, 23). Direct evidence for the unique influence of the adrenal stroma comes from orthotopic neuroblastoma models: intra-adrenal tumors are more aggressive and show a distinct immune infiltrate compared to subcutaneous tumors, supporting a possible role for the adrenal microenvironment in shaping tumor–immune phenotypes (24).

### 2.2 Acellular components: extracellular matrix remodeling and metabolic stress

The extracellular matrix (ECM) in adrenal tumors may undergo increased crosslinking and stiffening, by extrapolation from other solid tumors, which can activate YAP/TAZ signaling and promote abnormal growth (25). In human ACC, deregulated hyaluronan metabolism has been observed, and hyaluronan fragments can contribute to immunosuppression by promoting PD-L1^+^ TAMs (18). Collagen fragments, as shown in non-adrenal systems, suppress T cell IFN-γ release via LAIR-1, a mechanism that may also operate in the adrenal TME (26). Metabolically, hypoxia, acidosis, and nutrient competition have been documented in ACC and are proposed to impair immune effector function, although direct functional validation in adrenal tumors remains limited (27, 28).

### 2.3 The defining feature: bidirectional hormone–immune crosstalk

**Figure 1:**
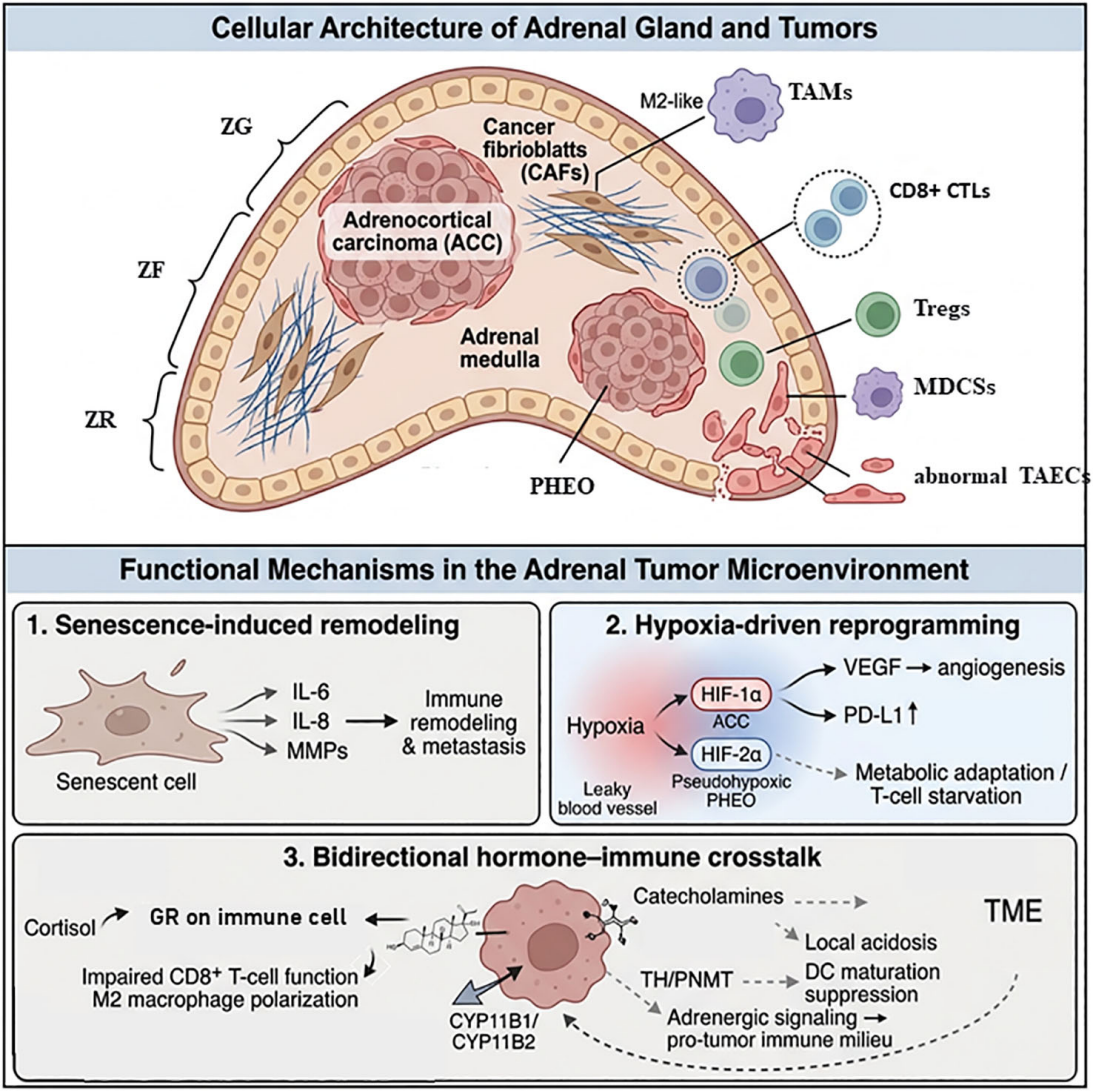
The cellular and functional landscape of the adrenal tumor microenvironment. This schematic uses a cross-section of the adrenal gland to illustrate the cellular components and key functional drivers that shape the tumor microenvironment (TME) in cortical and medullary tumors. Solid arrows indicate mechanisms supported by direct evidence from human adrenal tumors or adrenal-specific preclinical models. Dashed arrows indicate hypothetical mechanisms or concepts extrapolated from other malignancies, which require future validation. Upper panel – cellular architecture. The adrenal cortex including zona glomerulosa (ZG), fasciculata (ZF), reticularis (ZR) and medulla are shown. Representative tumors are depicted: an adrenocortical carcinoma (ACC) arising from the cortex and a pheochromocytoma (PHEO) arising from the medulla. The following cell types, all documented in human adrenal tumor specimens, are depicted as solid-outlined icons: The tumor nests are surrounded by a dynamic ecosystem including M2-polarised tumor-associated macrophages (TAMs)- confirmed in human APA, CPA, ACC, and PPGL, cancer-associated fibroblasts (CAFs) depositing a dense fibrotic extracellular matrix (ECM), CD8^+^ cytotoxic T lymphocytes (CTLs) – shown with dotted circles to reflect their variable abundance (sparse in ACC, more numerous in some PPGL subtypes); regulatory T cells (Tregs), myeloid-derived suppressor cells (MDSCs), and abnormal tumor-associated endothelial cells (TAECs) forming disorganized vessels. All these cell types have been documented in human adrenal tumors. Lower panel – functional mechanisms. • Senescence-induced remodeling: A subset of tumor and stromal cells undergo senescence, secreting a senescence-associated secretory phenotype (SASP; IL-6, IL-8, MMPs). This remodels the immune infiltrate in a sex-dimorphic manner and promotes metastasis (evidence from adrenal-specific preclinical models). • Hypoxia-driven reprogramming: Hypoxic regions stabilize HIF-1α (in ACC) or HIF-2α (in pseudohypoxic PHEO). HIF activation drives VEGF-mediated angiogenesis and upregulates PD-L1 (human correlative data). Downstream effects on T cell metabolism (glycolytic shift) remain hypothetical. • Bidirectional hormone–immune crosstalk: In cortical tumors, glucocorticoids signal through the glucocorticoid receptor (GR) on immune cells, impairing effector T cell function and promoting M2 polarization (human evidence). In medullary tumors, catecholamines are hypothesized to acidify the microenvironment, suppress dendritic cell maturation, and engage adrenergic signalling (extrapolated from non-adrenal models). Conversely, TME-derived factors may modulate steroidogenic enzymes (CYP11B1, CYP11B2) and catecholamine-synthesizing enzymes (TH, PNMT), but direct evidence in adrenal tumors is lacking. This direct, bidirectional endocrine–immune dialogue is a defining hallmark of the adrenal TME and is not typically found in non-endocrine solid tumors..

A distinguishing characteristic of the adrenal TME is that stromal and ECM components can influence hormone synthesis. In PPGL, single-cell analysis has identified a multifunctional pheochromocyte co-expressing POMC, CRH, and ACTH, suggesting that the TME may facilitate neuroendocrine transdifferentiation (29). In cortisol-producing adenomas, local glucocorticoid excess creates an immunosuppressive milieu with shifted macrophage polarization, directly linking hormonal output to immune sculpting (7). Reciprocally, CAF-derived cytokines can modulate steroidogenic enzyme expression, as shown *in vitro* (22). This bidirectional hormone–microenvironment network is a defining hallmark of adrenal tumors and provides a conceptual framework for understanding how endocrine activity and immune evasion co-evolve (**Table 1**; **Figure 1**).

**Table 1:** Comparison of TME features across adrenal tumor subtype.

Feature	APA	CPA	ACC	PHEO/PPGL
Immune Infiltrate	M2 macrophages, B cells, some CD8+ T cells	Shifted CD4/CD8 ratio, altered macrophage polarization	Relatively immune-depleted, M2 TAMs, MDSCs	CD8+ T cells, CD68+ macrophages; M2 in SDHx mutants
Key Immunosuppressive Factor	KCNJ5 mutation effects, ion homeostasis	Local cortisol excess, GR signaling	Senescence (SASP), hypoxia, GR signaling	Catecholamine metabolites, HIF-2α signaling
Angiogenesis	VWF+ endothelial cells	Not prominent	Hypoxia-driven, abnormal vasculature	Highly angiogenic, HIF-1α/2α-driven
Stroma/ECM	Hedgehog-driven remodeling	Remodeling by CAFs	Dense fibrotic ECM, CAF-driven	S100+ sustentacular cells, neural component
Clinical Significance	Model for benign tumor immune evasion; potential recurrence risk	Explains metabolic comorbidities; GR targeted therapy	Prognostic TME subtypes; predicts immunotherapy response	Identifies aggressive potential; correlates with PASS/GAPP

## 3 The microenvironment of adrenocortical tumors

### 3.1 Aldosterone-producing adenoma: immune landscape and its clinical context

Aldosterone-producing adenoma (APA) is a benign tumor whose primary clinical significance lies in endocrine hypertension and its cardiovascular sequelae; surgical resection or mineralocorticoid receptor blockade remains the cornerstone of management (6). Nevertheless, single-nucleus and spatial transcriptomics have revealed an immunosuppressive TME in APA, characterized by M2-polarized macrophages and specific endothelial subsets (14, 30). These tumors can be subtyped into APA-I (zona glomerulosa signature) and APA-II (zona fasciculata/reticularis signature), with distinct metabolic profiles (31). APA is thought to arise from aldosterone-producing cell clusters (APCCs), and somatic KCNJ5 mutations are associated with two cell states linked to oxidative stress pathways (30, 32). How this immune landscape relates to the pathogenesis of aldosterone excess or the rare progression to malignancy is not yet clear. By extrapolation from other tumor models, intra-tumoral potassium and potassium channel activity (e.g., Kir2.1, Eag1) can influence macrophage polarization (33, 34), but whether such ion–immune crosstalk operates in APA remains an open hypothesis. In benign APA, TME characterization is therefore valuable primarily for understanding endocrine pathophysiology and for identifying biomarkers of aggressive potential, rather than for immediate immunotherapeutic application.

### 3.2 Cortisol-producing adenoma: local glucocorticoid excess sculpts the immune microenvironment

In cortisol-producing adenoma (CPA), the defining feature of the TME is the local accumulation of glucocorticoids, which engages the glucocorticoid receptor (GR) on infiltrating immune cells. This local cortisol excess has been observed in human adrenal tumor cohorts to correlate with altered immune infiltration, including effects on CD8^+^ T cells, regulatory T cells, NK cells, and macrophages, as well as upregulation of PD-L1 (7, 35). A GR activity signature has been identified in ACC that distinguishes subtypes with different immune landscapes, suggesting a conserved mechanism through which local steroidogenesis can shape immune states (36). Paradoxically, disorganized glucocorticoid production *in situ* may create microdomains of immunosuppression within an otherwise inflamed tumor bed, impairing local anti-tumor immunity (37). Thus, even in a benign adenoma, the cortisol-laden TME offers a unique model for studying how chronic local glucocorticoid exposure drives immune adaptation and potentially contributes to systemic metabolic comorbidities.

These insights from benign cortical adenomas set the stage for understanding adrenocortical carcinoma, where the same principles of steroid-driven immunosuppression are amplified and combined with additional layers of immune escape.

### 3.3 Adrenocortical carcinoma: why does it remain relatively resistant to immunotherapy?

Despite identifiable immune and molecular subtypes, ACC responds poorly to current immunotherapies. We propose that this resistance arises from a convergence of non-redundant immunosuppressive mechanisms.

#### 3.3.1 An immune-depleted phenotype and cortisol/GR-mediated immunosuppression

A single-nucleus atlas of 38 human adrenal tumors has revealed that the ACC microenvironment is relatively immune-depleted, with T cells and other immune populations combining into ecotypes that link steroid differentiation with immunosuppressive signatures, such as exhausted T cells co-localizing with a fasciculata steroid program (38). TME-based scoring identifies a “high-score” subtype (Subtype 2) with stronger immune signatures, yet this does not translate into consistent immunotherapy responses (17). Immunogenomic profiling has further refined distinct immune-specific ACC subtypes (39). Autonomous cortisol production, present in 40–60% of ACC, creates an immunosuppressive milieu through GR signaling: in human ACC, a GR activity signature correlates with immune cell infiltration, but the simultaneous presence of exhausted T cells and M2-polarized macrophages suggests that the infiltrate is functionally compromised (36). Disorganized *in situ* steroidogenesis may generate microdomains of profound immunosuppression even when overall immune infiltration appears high (37).

#### 3.3.2 Low to moderate tumor mutational burden and limited neoantigenicity

ACC generally exhibits low to moderate tumor mutational burden (TMB). In the phase II pembrolizumab trial, TMB was not associated with response (40). Mismatch repair deficiency occurs in approximately 14% of ACC (mainly MSH6 loss), but most dMMR tumors lack concomitant microsatellite instability, and dMMR status was not predictive of immune checkpoint inhibitor response (41). Thus, the neoantigen landscape of ACC rarely provides a strong endogenous immune stimulus.

#### 3.3.3 M2-polarized TAMs, MDSCs, and hypoxia-driven immune exclusion

ACC is enriched for M2-polarized TAMs and myeloid-derived suppressor cells (MDSCs) that physically and functionally exclude effector T cells (17, 42). A hypoxia-based risk score has been developed for ACC that stratifies prognosis, and hypoxic regions show upregulation of HIF-1α, VEGF, and metabolic reprogramming, contributing to immune exclusion (28). These features collectively generate a microenvironment hostile to T cell infiltration and function.

#### 3.3.4 Cellular senescence and sex-dimorphic immune remodeling

Accumulation of senescent cells has been observed in ACC and, in preclinical models, triggers a senescence-associated secretory phenotype (SASP) that remodels the immune infiltrate in a sex-dimorphic manner, actively facilitating metastatic spread (43). This represents a non-canonical axis of immunosuppression that may not be overcome by checkpoint blockade alone.

#### 3.3.5 Treatment-related confounding: mitotane

Mitotane, the standard-of-care for advanced ACC, is a potent inducer of CYP3A4, raising the possibility of pharmacokinetic interactions with immune checkpoint inhibitors, although this remains pharmacologically unestablished (44–48). This potential confounding factor adds another layer of complexity to interpreting immunotherapy trial results.

**Figure 2:**
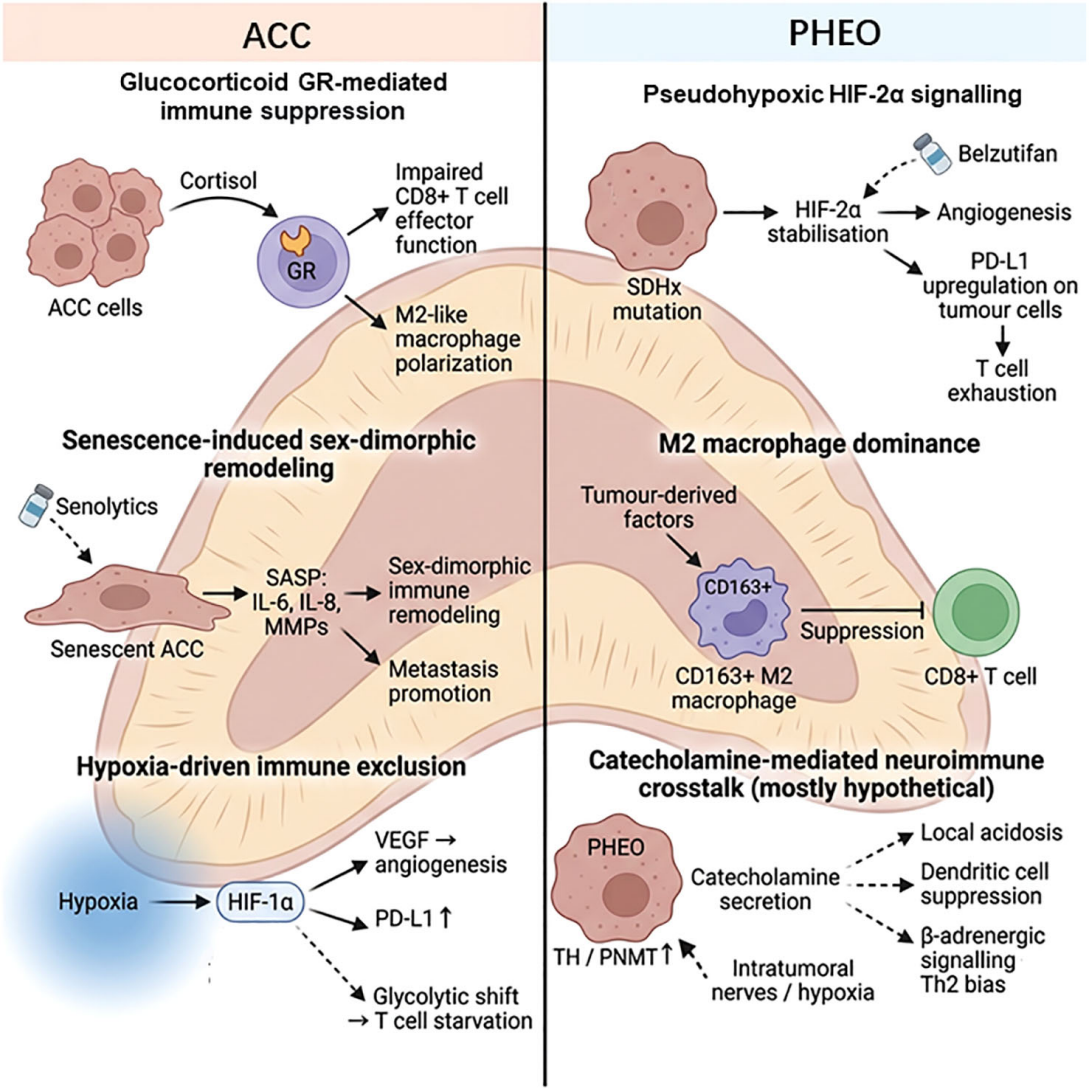
Distinct immunosuppressive circuits define the tumor microenvironments of adrenocortical carcinoma and pheochromocytoma. This side-by-side comparison uses a cross-section of the adrenal gland for anatomical context. Solid arrows represent mechanisms with direct evidence from human adrenal tumor studies or adrenal-specific preclinical models. Dashed arrows represent hypothetical mechanisms or future therapeutic concepts lacking direct adrenal validation. Left panel – Adrenocortical carcinoma (ACC). • Glucocorticoid-GR axis: Autonomous cortisol production signals through GR on immune cells. Although GR activity correlates with immune infiltration, disorganized local steroid production impairs CD8^+^ T cell function and promotes M2-like macrophage polarization (correlative human data; causal relationship not yet proven). This axis uniquely positions local, autonomous steroidogenesis as a dominant, tumor-intrinsic mode of immunosuppression in ACC. • Senescence-SASP axis: Senescent tumor cells secrete IL-6, IL-8, and MMPs, leading to sex-dimorphic immune remodelling and metastasis (adrenal-specific preclinical model). • Hypoxia-HIF-1α axis: Hypoxia stabilizes HIF-1α, which induces VEGF-mediated angiogenesis and PD-L1 upregulation (human correlative data). The proposed glycolytic shift that starves T cells remains hypothetical. Right panel – Pheochromocytoma/paraganglioma (PHEO/PPGL). • Pseudohypoxic HIF-2α axis: In SDHx-mutant and other Cluster 1 tumors, HIF-2α stabilization drives angiogenesis and directly upregulates PD-L1 on tumor cells, contributing to T cell exhaustion (human correlative evidence). • M2 macrophage dominance: The PHEO TME is enriched for CD163^+^ M2-polarised TAMs, which inversely correlate with CD8^+^ T cell infiltration and higher M2/CD8 ratio in pseudohypoxic subtypes associate with higher metastatic risk (human immunohistochemical data). • Catecholamine-mediated crosstalk (hypothetical): Catecholamine excess is hypothesized to acidify the microenvironment, suppress dendritic cell maturation, and engage β-adrenergic signalling to promote a pro-tumor immune state. Reciprocal signals from intratumoral nerves and hypoxic zones may sustain catecholamine synthesis by upregulating TH and PNMT. All mechanisms in this circuit are drawn with dashed arrows, as they are extrapolated from non-adrenal models and require direct validation in PPGL. If confirmed, this crosstalk would represent a unique, catecholamine-driven neuro-endocrine-immune axis specific to chromaffin-cell tumors..

Collectively, these mechanisms—steroid-mediated suppression, low neoantigenicity, myeloid-driven exclusion, hypoxia, senescence, and possibly drug interactions—provide a plausible explanation for the limited efficacy of single-agent immunotherapy in ACC. They also point to rational combination targets, which we discuss in Section 6 (**Figure 2**).

## 4 The microenvironment of pheochromocytoma and paraganglioma

### 4.1 Pseudohypoxic and kinase-signaling subtypes define distinct immune landscapes

Multi-omics and single-cell genomics have identified up to seven molecular subtypes of PPGL, driven by mutations in hypoxia-inducible factors, Krebs cycle genes, kinases, and WNT signaling, as well as by chromaffin differentiation states (11, 49, 50). Pseudohypoxic tumors (Cluster 1), particularly SDHx-mutant PPGL, are enriched for M2-like macrophages and display an immune-cold phenotype, with low CD8^+^ T cell infiltration (50). In contrast, kinase-signaling subtypes (Cluster 2) exhibit more heterogeneous, occasionally inflamed infiltrates (50). Transcriptome-guided analysis of ligand–receptor crosstalk has revealed subtype-specific communication networks governing immune cell recruitment and polarization (51). Metastatic progression typically requires secondary alterations in TERT or ATRX, which act in concert with a remodeled microenvironment (49, 52). Cancer stemness indices are higher in metastatic PPGL and are associated with an immunosuppressive phenotype, suggesting a link between dedifferentiation and immune evasion (53).

### 4.2 Catecholamine metabolism and microenvironment crosstalk

Catecholamine excess, the hormonal hallmark of PPGL, can directly influence the immune microenvironment. In human PHEO, deficient expression of catecholamine-synthesizing enzymes (PNMT, TH) has been associated with more aggressive histological features and larger tumor size (21). While direct functional evidence in PPGL remains limited, by extrapolation from other models, catecholamine metabolites may contribute to local acidosis, which suppresses T cell function and promotes invasion (54). Catecholamines can also signal through β-adrenergic receptors on immune cells, suppressing type I interferon responses and favoring a Th2-skewed, pro-tumor milieu (50). These data suggest that catecholamine output not only drives systemic symptoms but also shapes a tumor-promoting immune state.

### 4.3 Immune phenotype and macrophage polarization

Immunohistochemical profiling of PPGL has revealed abundant CD68^+^ macrophages and, in some series, CD163^+^ monocytes that may outnumber sustentacular cells (21, 55). High CD163 protein expression has been associated with SDH mutations, linking macrophage polarization directly to oncogenic genotype (55). A negative correlation has been observed between the M2 polarization ratio (CD163/CD68) and SSTR2A expression, and a positive correlation between HIF-2α and PD-L1 expression; co-expression of HIF-2α and PD-L1 was found in 15.6% of patients, identifying a subset that might rationally be targeted with combined HIF-2α inhibition and immunotherapy (56). MAML3-fusion-positive PPGL overexpresses PD-L1 and CD40, potentially representing another immunologically distinct subset (57, 58) (**Figure 2**).

## 5 Key pathways modulating hormone secretion from the microenvironment

### 5.1 Cytokine-mediated regulation of steroidogenic enzymes

Inflammatory cytokines such as IL-1β and TNF-α have been shown, in non-adrenal endocrine studies, to acutely stimulate cortisol secretion and chronically modulate the expression of steroidogenic enzymes (CYP11B1, CYP11B2, CYP17A1) via NF-κB and AP-1 pathways (59, 60). TAM-derived TGF-β suppresses CD8^+^ T cell function and, in preclinical adrenal models, disrupts StAR and CYP11A1 expression, leading to dysregulated steroidogenesis (61–63). In rare ectopic ACTH/CRH-secreting PHEOs, single-cell analysis has identified a multifunctional pheochromocyte that may directly stimulate cortical cortisol synthesis through a paracrine loop (29). Collectively, these findings suggest that the adrenal TME harbors cytokine circuits capable of fine-tuning hormone output, although direct demonstration of such modulation in human adrenal tumor tissue remains limited (**Table 2**).

**Table 2:** Summary of key single-cell and spatial omics studies in adrenal tumors.

Tumor type	Sample size (n)	Technology	Key findings	References
APA, normal adrenal	12 APAs, 6 healthy	snRNA-seq, spatial transcriptomics	Identified 6 adenoma-specific clusters, M2 macrophages and specific endothelial subsets	(14)
ACC, normal adrenal	38 human samples	snRNA-seq	Discovered ecotypes combining steroid and microenvironment cell signatures; revealed relative immune depletion in ACC	(38)
Ectopic ACTH/CRH PHEO	1 case	scRNA-seq	Discovered novel bifunctional pheochromocyte (POMC+CRH+)	(29)
PPGL subtypes	Multiple datasets	Transcriptomic (Bulk/sc)	Mapped subtype-specific ligand-receptor crosstalk between tumor and stroma	(51)

### 5.2 Hypoxia-inducible factor-driven reprogramming

Hypoxia stabilizes HIFs, which remodel both metabolism and endocrine function. A hypoxia-based risk score has been developed for ACC and suggests that HIF activation may influence tumor functional status (28). In PPGL, pseudohypoxic signaling is intimately tied to the tumor’s catecholaminergic phenotype (49). While HIF-mediated hormone reprogramming in adrenal tumors is an emerging concept, by extrapolation from other models, hypoxia may modulate the expression of steroidogenic enzymes, warranting further investigation.

### 5.3 Exosomal miRNA-mediated transcellular regulatory networks

Cancer-associated fibroblasts secrete exosomes that transfer signaling molecules and influence tumor behavior, as established in multiple cancer types (64). In adrenal tumors, circulating extracellular vesicle-associated microRNAs are differentially expressed between non-functioning and cortisol-producing adrenocortical tumors, suggesting that exosomal cargo may reflect functional status (65). However, evidence that exosomal miRNAs directly modulate steroidogenic enzyme expression is currently lacking; this axis remains a hypothesis requiring experimental validation.

These pathways illustrate how the TME feeds back on hormone secretion, revealing potential therapeutic targets. This insight informs the immunotherapeutic opportunities and combination strategies discussed below.

## 6 Immunotherapeutic opportunities and combination strategies

The evidence supporting immunotherapeutic strategies in adrenal tumors spans a wide spectrum, from clinical trial data to hypotheses extrapolated from other malignancies. To guide interpretation, we organize current strategies according to the Level A–D evidence classification introduced in Section 1.3.

### 6.1 Level A evidence: strategies supported by human adrenal tumor studies

#### 6.1.1 Immune checkpoint blockade

The most mature immunotherapeutic data in adrenal tumors concern immune checkpoint inhibitors (ICIs). In adrenocortical carcinoma (ACC), a phase II study of pembrolizumab in 39 patients reported an objective response rate (ORR) of 23% and a disease control rate of 52%, with responses occurring in both MSI-H/dMMR and MSS tumors (40). Avelumab in the JAVELIN trial achieved an ORR of 6.0% in 50 patients with platinum-treated metastatic ACC; PD-L1 positivity (≥5%) was associated with a numerically higher ORR (16.7% vs. 3.3%), though this difference was not statistically significant (66). A 2024 meta-analysis of 20 studies (250 patients) confirmed modest activity, with a pooled ORR of 14% (67). In pheochromocytoma and paraganglioma (PPGL), a phase II trial of pembrolizumab reported a clinical benefit rate of 75% and non-progression at 27 weeks in 43% of evaluable patients (68). Retrospective data suggest an ORR of approximately 9%, with MAML3-fusion-positive tumors, which overexpress PD-L1 and CD40, identified as a potentially responsive subset (69).

#### 6.1.2 HIF-2α inhibition in pseudohypoxic PPGL

Belzutifan, an HIF-2α inhibitor, has demonstrated clinical activity in advanced PPGL (70). In PPGL, co-expression of HIF-2α and PD-L1 has been documented in 15.6% of patients (56), suggesting that combined HIF-2α inhibition and PD-1 blockade may be a rational strategy for this molecularly defined subset. However, this combination has not been tested in any adrenal tumor clinical trial or preclinical model. Therefore, while the single−agent HIF−2α inhibitor is supported by Level A data, the combination strategy currently rests only on correlative biomarker evidence and mechanistic plausibility, placing it at Level C (endocrine−immune mechanism not directly validated in adrenal tumors).

#### 6.1.3 Biomarker studies guiding patient selection

Several Level A studies have evaluated biomarkers of response or resistance. PD-L1 expression is elevated in PPGL compared to normal adrenal medulla but is lower in pseudohypoxic subtypes (SDHB, VHL) than in kinase-signaling or sporadic tumors, indicating genotype-dependent expression (69). In ACC, PD-L1 expression was not predictive of pembrolizumab response (40). Tumor mutational burden (TMB) is generally low to moderate in ACC and was not associated with ICI response (40). Mismatch repair deficiency occurs in approximately 14% of ACC (mainly MSH6 loss), but most dMMR tumors lack concomitant microsatellite instability, and dMMR status did not predict immunotherapy benefit (41). These data highlight both the potential and the limitations of tissue-based biomarkers for immunotherapy in adrenal tumors.

### 6.2 Level B evidence: strategies validated in adrenal-specific preclinical models

#### 6.2.1 Senolytics in ACC

Cellular senescence has been identified in human ACC tissue, and in a genetically engineered mouse model of ACC, pharmacological clearance of senescent cells reversed sex-dimorphic immune remodeling and reduced metastatic progression (43). This provides adrenal-specific preclinical proof-of-concept that targeting the senescence-associated secretory phenotype (SASP) can reshape the immune microenvironment. Clinical translation of senolytic approaches in ACC may therefore represent a strategy with direct preclinical validation.

### 6.3 Level C evidence: strategies grounded in endocrine–immune mechanisms but not directly tested in adrenal tumors

#### 6.3.1 Glucocorticoid receptor antagonism

Endogenous glucocorticoid signaling, acting through the glucocorticoid receptor (GR), suppresses CD8^+^ T cell effector function and promotes M2-like macrophage polarization, as established in non-adrenal systems (16). In human ACC, a GR activity signature has been shown to correlate with immune cell infiltration, supporting the biological relevance of this axis (36). However, the concept of pharmacological GR blockade to reverse cortisol-mediated immune suppression has not yet been tested in adrenal-specific models or clinical trials. GR antagonism therefore represents a strategy with a strong endocrine-mechanistic rationale (Level C) and supportive correlative human data (Level A), but it lacks direct experimental validation in adrenal tumors.

#### 6.3.2 Targeting catecholamine–adrenergic signaling

Catecholamines can signal through β-adrenergic receptors on immune cells to suppress type I interferon responses and promote a Th2-skewed milieu (50). In PPGL, deficient expression of catecholamine-synthesizing enzymes has been associated with more aggressive features (21), suggesting that adrenergic blockade could modulate the immune microenvironment. This strategy remains mechanistically plausible (Level C) but has not been functionally tested in PPGL models.

### 6.4 Level D evidence: strategies extrapolated from other malignancies

Several TME-modulating strategies that have shown promise in other cancers remain entirely hypothetical in adrenal tumors.

#### 6.4.1 CSF-1R blockade

Colony-stimulating factor 1 receptor (CSF-1R) inhibition can deplete immunosuppressive TAMs and enhance anti-tumor immunity in non-adrenal preclinical models (71). Given the prominence of M2-like TAMs in ACC and pseudohypoxic PPGL, CSF-1R blockade may represent a rational hypothesis for future testing, but no adrenal-specific evidence currently exists.

#### 6.4.2 Macrophage repolarization via ion channel modulation

Inhibition of the Kir2.1 potassium channel has been shown to repolarize M2-like TAMs toward an M1-like phenotype in other tumor models (33). Similarly, the voltage-gated potassium channel Eag1 (Kv10.1) is overexpressed in many cancers and has been proposed as a therapeutic target (34). However, the relevance of these ion-channel-based macrophage reprogramming strategies to adrenal tumors has not been investigated.

#### 6.4.3 CAF-targeted and vascular normalization strategies

Cancer-associated fibroblasts (CAFs) contribute to immune exclusion in multiple cancer types (22), and anti-angiogenic agents can transiently normalize tumor vasculature, potentially improving immune cell infiltration. While the angiogenic phenotype of PPGL and the presence of CAFs in ACC are documented, strategies to therapeutically target CAFs or normalize the vasculature to enhance immunotherapy remain extrapolations from other malignancies (Level D).

#### 6.4.4 Additional considerations: treatment-related confounding

Mitotane, the standard therapy for advanced ACC, is a potent inducer of CYP3A4 and could theoretically alter the pharmacokinetics of certain immune checkpoint inhibitors, although this interaction has not been formally studied (44–48). In PPGL, the impact of antihypertensive medications on immune function is unknown. These potential confounders underscore the need for careful pharmacodynamic assessment in future immunotherapy trials.

Collectively, this evidence landscape reveals that the vast majority of proposed TME-targeted interventions for adrenal tumors remain hypothetical, with only ICIs and HIF-2α inhibition supported by Level A clinical data. Even these strategies show only modest efficacy, likely due to the multiple layers of immunosuppression detailed in Sections 3 and 4. The path forward will require rigorous preclinical validation of candidate combination regimens in adrenal-specific models, followed by biomarker-driven clinical trials that account for the unique hormonal and molecular heterogeneity of these tumors (**Table 3**).

**Table 3:** Immunotherapeutic and TME-targeting strategies: evidence levels.

Therapeutic strategy	Tumor type	Evidence level	Key findings / status	References
Anti-PD-1 (pembrolizumab)	ACC	Level A	ORR 23%, DCR 52%; responses in both MSI-H/MMR-D and MSS tumors; TMB and PD-L1 not predictive	(40)
Anti-PD-L1 (avelumab)	ACC	Level A	ORR 6.0% in platinum-treated metastatic ACC (JAVELIN trial)	(66)
ICIs broadly	ACC	Level A	Pooled ORR 14% (95% CI 10–19%); median OS 13.9 months (20 studies, 250 patients)	(67)
Anti-PD-1 (pembrolizumab)	PPGL	Level A	Clinical benefit rate 75%; non-progression at 27 weeks 43% (3/7 evaluable)	(68)
ICIs broadly	PPGL	Level A	ORR ~9%; PD-L1+ MAML3-related tumors may be a responsive subset	(57, 58, 69)
HIF-2α inhibitor (belzutifan)	Pseudohypoxic PPGL	Level A	Demonstrated clinical activity in advanced PPGL; represents first targeted therapy for pseudohypoxic subset	(70)
HIF-2α inhibitor (belzutifan) + anti-PD-1	Pseudohypoxic PPGL	Level C	HIF-2α and PD-L1 co-expressed in 15.6% of PPGLs; HIF-2α inhibition may reverse pseudohypoxia-driven immunosuppression and sensitize to PD-1 blockade	(56, 70)
β-adrenergic receptor blockade	PPGL	Level C	Catecholamines may suppress type I interferon responses and promote Th2-skewed milieu; not functionally tested in PPGL models	(20, 50)
Senolytics	ACC	Level B	Clearance of senescent cells reversed sex-dimorphic immune remodeling and reduced metastasis	(43)
GR antagonist + ICIs	ACC	Level C	GR activity signature correlates with immune infiltration and prognosis; GR antagonism may reverse cortisol-mediated immune paralysis and synergize with ICIs	(16, 36)
CAF-targeted therapy	ACC / PPGL	Level D	CAFs contribute to immune exclusion in multiple cancers; not investigated in adrenal tumors	(22)
Vascular normalization (anti-angiogenics)	PPGL / ACC	Level D	May improve immune cell infiltration; extrapolated from other malignancies; no adrenal-specific evidence	(23)
CSF-1R blockade (TAM depletion)	ACC / PPGL	Level D	Depletes pro-tumor TAMs in non-adrenal preclinical models; not directly tested in adrenal tumors	(71)
Kir2.1 inhibition (M2→M1 repolarization)	APA (speculative)	Level D	Can repolarize M2-TAMs to M1-like phenotype in non-adrenal models; remains speculative in adrenal tumors	(33)

## 7 Challenges, limitations, and future directions

### 7.1 Key obstacles to clinical translation

Translation of adrenal TME insights is hindered by disease rarity, which limits cohort sizes and statistical power in most immunogenomic studies (40, 66–68). Beyond sample size, biological variables specific to endocrine tumors—hormone secretion status, prior mitotane or antihypertensive therapy, genetic background, and sampling site—are inconsistently controlled, yet can profoundly influence immune infiltration (16, 36, 43). Sex-dimorphic immune remodeling and adrenal aging introduce additional, often unaccounted, sources of variation (3, 43).

### 7.2 Technical limitations of current approaches

Bulk transcriptomic deconvolution is limited by reference-cell bias and the difficulty of distinguishing resident adrenal immune cells from tumor-infiltrating populations. Single-cell and spatial omics, while transformative, carry their own caveats. Enzymatic dissociation may preferentially lose fragile immune and stromal populations, and stress-response genes can be induced during tissue processing. Spatial transcriptomics preserves tissue architecture but often lacks single-cell resolution, and FFPE-based platforms may differ from fresh-tissue approaches (72, 73). Furthermore, adrenal tumors contain steroidogenic and chromaffin cells with distinctive metabolic and transcriptional programs, complicating normalization, cell-type annotation, and cross-study integration (38). Omics-derived immune states should therefore be regarded as hypotheses requiring orthogonal validation by multiplex immunohistochemistry, flow cytometry, functional assays, and clinical correlation.

### 7.3 Mechanistic gaps and research priorities

Several fundamental questions remain open. Causal relationships between local hormone production and immune phenotypes have not been directly demonstrated in human adrenal tumors; most evidence remains correlative. Whether immunosuppressive features of benign adenomas actively drive tumor maintenance or are bystander effects of hormone excess is unknown. The cell-of-origin for cancer-associated fibroblasts in the adrenal gland is undefined, and the impact of standard therapies (mitotane, α/β-blockade) on the TME has not been systematically studied. Addressing these gaps will require multi-center cohorts with standardized annotation of hormone secretion, genotype, and treatment history; longitudinal sampling; integrated spatial multi-omics; and functional validation in adrenal-relevant models, including organoid co-cultures that preserve steroidogenic capacity. Given the rarity of these diseases, biomarker-driven, subtype-specific combination trials—such as GR antagonism in cortisol-producing ACC or HIF-2α inhibition plus PD-1 blockade in pseudohypoxic PPGL—will require innovative, collaborative trial designs.

## References

[1] Ozisik G Achermann JC Meeks JJ Jameson JL . SF1 in the development of the adrenal gland and gonads. Horm Res. (2003) 59(Suppl 1):94–98. doi: 10.1159/000067831 12566727

[2] Relav L Doghman-Bouguerra M Ruggiero C Muzzi JCD Figueiredo BC Lalli E . Steroidogenic factor 1, a Goldilocks transcription factor from adrenocortical organogenesis to malignancy. Int J Mol Sci. (2023) 24(4). doi: 10.3390/ijms24043585 36835002 PMC9959402

[3] Warde KM Smith LJ Basham KJ . Age-related changes in the adrenal cortex: insights and implications. J Endocr Soc. (2023) 7(9):bvad097. doi: 10.1210/jendso/bvad097 37564884 PMC10410302

[4] Wood MA Hammer GD . Adrenocortical stem and progenitor cells: unifying model of two proposed origins. Mol Cell Endocrinol. (2011) 336(1-2):206–212. doi: 10.1016/j.mce.2010.11.012 21094677 PMC3397472

[5] Chu Y Setayesh J Dumontet T Krumeich L Werner J Moretti IF . Adrenocortical stem cells in health and disease. Nat Rev Endocrinol. (2025) 21(8):464–481. doi: 10.1038/s41574-025-01091-2 40065108 PMC13177385

[6] Funder JW Carey RM Mantero F Murad MH Reincke M Shibata H . The management of primary aldosteronism: case detection, diagnosis, and treatment: an Endocrine Society clinical practice guideline. J Clin Endocrinol Metab. (2016) 101(5):1889–1916. doi: 10.1210/jc.2015-4061 26934393

[7] Kitawaki Y Nakamura Y Kubota-Nakayama F Yamazaki Y Miki Y Hata S . Tumor microenvironment in functional adrenocortical adenomas: immune cell infiltration in cortisol-producing adrenocortical adenoma. Hum Pathol. (2018) 77:88–97. doi: 10.1016/j.humpath.2018.03.016 29596893

[8] Mete O Erickson LA Juhlin CC de Krijger RR Sasano H Volante M . Overview of the 2022 WHO classification of adrenal cortical tumors. Endocr Pathol. (2022) 33(1):155–196. doi: 10.1007/s12022-022-09710-8 35288842 PMC8920443

[9] Else T Kim AC Sabolch A Raymond VM Kandathil A Caoili EM . Adrenocortical carcinoma. Endocr Rev. (2014) 35(2):282–326. doi: 10.1210/er.2013-1029 24423978 PMC3963263

[10] Fassnacht M Dekkers OM Else T Baudin E Berruti A de Krijger R . European Society of Endocrinology clinical practice guidelines on the management of adrenocortical carcinoma in adults, in collaboration with the European Network for the Study of Adrenal Tumors. Eur J Endocrinol. (2018) 179(4):G1–G46. doi: 10.1530/eje-18-0608 30299884

[11] Fishbein L Leshchiner I Walter V Danilova L Robertson AG Johnson AR . Comprehensive molecular characterization of pheochromocytoma and paraganglioma. Cancer Cell. (2017) 31(2):181–193. doi: 10.1016/j.ccell.2017.01.001 28162975 PMC5643159

[12] Liu NM Sholevar CJ Karimzadeh M Uppuluri J Van Dongen C Graves CE . The immune biology of the adrenal gland microenvironment and its role in metastatic progression. Int J Mol Sci. (2026) 27(3). doi: 10.3390/ijms27031153 41683577 PMC12897121

[13] Varghese J Habra MA . Update on adrenocortical carcinoma management and future directions. Curr Opin Endocrinol Diabetes Obes. (2017) 24(3):208–214. doi: 10.1097/med.0000000000000332 28277340

[14] Altieri B Secener AK Sai S Fischer C Sbiera S Arampatzi P . Single-nucleus and spatial transcriptome reveal adrenal homeostasis in normal and tumoural adrenal glands. Clin Transl Med. (2024) 14(8):e1798. doi: 10.1002/ctm2.1798 39167619 PMC11338279

[15] Huang J Qin F Lai X Yang T Yu J Wei C . Exploring heterogeneity of tumor immune cells and adrenal cells in aldosterone-producing adenomas using single-cell RNA-seq and investigating differences by sex. Heliyon. (2023) 9(3):e14357. doi: 10.1016/j.heliyon.2023.e14357 36942259 PMC10024085

[16] Acharya N Madi A Zhang H Klapholz M Escobar G Dulberg S . Endogenous glucocorticoid signaling regulates CD8(+) T cell differentiation and development of dysfunction in the tumor microenvironment. Immunity. (2020) 53(3):658–671.e6. doi: 10.1016/j.immuni.2020.08.005 32937153 PMC7682805

[17] Lai G Liu H Deng J Li K Zhang C Zhong X . The characteristics of tumor microenvironment predict survival and response to immunotherapy in adrenocortical carcinomas. Cells. (2023) 12(5). doi: 10.3390/cells12050755 36899891 PMC10000893

[18] Donelan W Dominguez-Gutierrez PR Kusmartsev S . Deregulated hyaluronan metabolism in the tumor microenvironment drives cancer inflammation and tumor-associated immune suppression. Front Immunol. (2022) 13:971278. doi: 10.3389/fimmu.2022.971278 36238286 PMC9550864

[19] WangY WilfahrtD JonkerP LontosK CaiC CameronB . Tumour interstitial fluid-enriched phosphoethanolamine suppresses T cell function. Nat Cell Biol. (2025) 27(5):835–846. doi: 10.1038/s41556-025-01650-9 40258951

[20] Globig AM Zhao S Roginsky J Maltez VI Guiza J Avina-Ochoa N . The beta(1)-adrenergic receptor links sympathetic nerves to T cell exhaustion. Nature. (2023) 622(7982):383–392. doi: 10.1038/s41586-023-06568-6 37731001 PMC10871066

[21] Gao X Yamazaki Y Pecori A Tezuka Y Ono Y Omata K . Histopathological analysis of tumor microenvironment and angiogenesis in pheochromocytoma. Front Endocrinol. (2020) 11:587779. doi: 10.3389/fendo.2020.587779 33244312 PMC7685215

[22] Mhaidly R Mechta-Grigoriou F . Role of cancer-associated fibroblast subpopulations in immune infiltration, as a new means of treatment in cancer. Immunol Rev. (2021) 302(1):259–272. doi: 10.1111/imr.12978 34013544 PMC8360036

[23] Leone P Malerba E Susca N Favoino E Perosa F Brunori G . Endothelial cells in tumor microenvironment: insights and perspectives. Front Immunol. (2024) 15:1367875. doi: 10.3389/fimmu.2024.1367875 38426109 PMC10902062

[24] Kroesen M Brok IC Reijnen D van Hout-Kuijer MA Zeelenberg IS Den Brok MH . Intra-adrenal murine TH-MYCN neuroblastoma tumors grow more aggressive and exhibit a distinct tumor microenvironment relative to their subcutaneous equivalents. Cancer Immunol Immunother. (2015) 64(5):563–572. doi: 10.1007/s00262-015-1663-y 25687736 PMC4412512

[25] Rubi-Sans G Nyga A Mateos-Timoneda MA Engel E . Substrate stiffness-dependent activation of Hippo pathway in cancer associated fibroblasts. Biomater Adv. (2025) 166:214061. doi: 10.1016/j.bioadv.2024.214061 39406156

[26] Vijver SV Singh A Mommers-Elshof E Meeldijk J Copeland R Boon L . Collagen fragments produced in cancer mediate T cell suppression through leukocyte-associated immunoglobulin-like receptor 1. Front Immunol. (2021) 12:733561. doi: 10.3389/fimmu.2021.733561 34691040 PMC8529287

[27] Deng X Liu X Zhao L . Multifunctional nanoplatforms for tumor microenvironment remodeling: toward precision and intelligent cancer therapy. Mater Today Bio. (2025) 35:102385. doi: 10.1016/j.mtbio.2025.102385 41142435 PMC12549778

[28] Deng Y Li H Fu J Pu Y Zhang Y Chen S . A hypoxia risk score for prognosis prediction and tumor microenvironment in adrenocortical carcinoma. Front Genet. (2022) 13:796681. doi: 10.3389/fgene.2022.796681 36583015 PMC9792869

[29] Zhang X Lian P Su M Ji Z Deng J Zheng G . Single-cell transcriptome analysis identifies a unique tumor cell type producing multiple hormones in ectopic ACTH and CRH secreting pheochromocytoma. Elife. (2021) 10. doi: 10.7554/elife.68436 34905486 PMC8719890

[30] Sun Z Jing C Tetti M Gong S Wei J Pang Y . Integrated transcriptomics reveals evolutionary trajectories and cell density-dependent mechanisms in aldosterone-producing adenomas. Adv Sci. (2026) 13(32):e05410. doi: 10.1002/advs.202505410 41562146 PMC13252614

[31] Gong S Sun N Meyer LS Tetti M Koupourtidou C Krebs S . Primary aldosteronism: spatial multiomics mapping of genotype-dependent heterogeneity and tumor expansion of aldosterone-producing adenomas. Hypertension. (2023) 80(7):1555–1567. doi: 10.1161/hypertensionaha.123.20921 37125608 PMC10330203

[32] Omata K Anand SK Hovelson DH Liu CJ Yamazaki Y Nakamura Y . Aldosterone-producing cell clusters frequently harbor somatic mutations and accumulate with age in normal adrenals. J Endocr Soc. (2017) 1(7):787–799. doi: 10.1210/js.2017-00134 29264530 PMC5686701

[33] Al-Sheikh U Kang L . Kir2.1 channel: macrophage plasticity in tumor microenvironment. Cell Metab. (2022) 34(11):1613–1615. doi: 10.1016/j.cmet.2022.10.009 36323230

[34] Toplak Z Hendrickx LA Abdelaziz R Shi X Peigneur S Tomasic T . Overcoming challenges of HERG potassium channel liability through rational design: Eag1 inhibitors for cancer treatment. Med Res Rev. (2022) 42(1):183–226. doi: 10.1002/med.21808 33945158

[35] Guo F Xu B Chen G Liu J Gao Q . Endogenous glucocorticoids and glucocorticoid receptor signaling: dual regulators of PD-1/PD-L1 immunotherapy efficacy in the tumor microenvironment. Chin Med J. (2026). doi: 10.1097/cm9.0000000000003956 41850767 PMC13384610

[36] Wu K Liu Z Liang J Zhu Y Wang X Li X . Discovery of a glucocorticoid receptor (GR) activity signature correlates with immune cell infiltration in adrenocortical carcinoma. J Immunother Cancer. (2023) 11(10). doi: 10.1136/jitc-2023-007528 37793855 PMC10551943

[37] Ishikawa Y Yamazaki Y Tezuka Y Omata K Ono Y Tokodai K . Histopathological analysis of tumor microenvironment in adrenocortical carcinoma: possible effects of in situ disorganized glucocorticoid production on tumor immunity. J Steroid Biochem Mol Biol. (2024) 238:106462. doi: 10.1016/j.jsbmb.2024.106462 38232786

[38] Jouinot A Martin Y Violon F Foulonneau T Bendjelal Y Calvet P . Impact of steroid differentiation on tumor microenvironment revealed by single-nucleus atlas of adrenal tumors. Nat Commun. (2025) 16(1):8860. doi: 10.1038/s41467-025-63912-2 41053133 PMC12501071

[39] Lu Q Nie R Luo J Wang X You L . Identifying immune-specific subtypes of adrenocortical carcinoma based on immunogenomic profiling. Biomolecules. (2023) 13(1). doi: 10.3390/biom13010104 36671489 PMC9855412

[40] Raj N Zheng Y Kelly V Katz SS Chou J Do RKG . PD-1 blockade in advanced adrenocortical carcinoma. J Clin Oncol. (2020) 38(1):71–80. doi: 10.1200/jco.19.01586 31644329 PMC7351334

[41] Altieri B Kircher S Herterich S Jahn A Teleanu MV Lippert J . Mismatch repair deficiency and microsatellite instability in adrenocortical carcinoma. ESMO Open. (2026) 11(2):106030. doi: 10.1016/j.esmoop.2025.106030 41650745 PMC12906187

[42] Ohmoto A Shigematsu Y Saito R Dobashi A Fujiwara Y Togashi Y . Prognosis and tumor microenvironment in pseudohypoxic pheochromocytoma/paraganglioma. Virchows Arch. (2025) 486(5):983–990. doi: 10.1007/s00428-024-04009-x 39694932

[43] Warde KM Smith LJ Liu L Stubben CJ Lohman BK Willett PW . Senescence-induced immune remodeling facilitates metastatic adrenal cancer in a sex-dimorphic manner. Nat Aging. (2023) 3(7):846–865. doi: 10.1038/s43587-023-00420-2 37231196 PMC11534150

[44] Zheng S Cherniack AD Dewal N Moffitt RA Danilova L Murray BA . Comprehensive pan-genomic characterization of adrenocortical carcinoma. Cancer Cell. (2016) 30(2):363. doi: 10.1016/j.ccell.2016.04.002 27505681

[45] Xu F Guan Y Zhang P Xue L Ma Y Gao M . Tumor mutational burden presents limiting effects on predicting the efficacy of immune checkpoint inhibitors and prognostic assessment in adrenocortical carcinoma. BMC Endocr Disord. (2022) 22(1):130. doi: 10.1186/s12902-022-01017-3 35568842 PMC9107278

[46] Bratslavsky G Sokol ES Daneshvar M Necchi A Shapiro O Jacob J . Clinically advanced pheochromocytomas and paragangliomas: a comprehensive genomic profiling study. Cancers. (2021) 13(13). doi: 10.3390/cancers13133312 34282751 PMC8268679

[47] Jin B Han W Guo J Tian J He S Gong Y . Initial characterization of immune microenvironment in pheochromocytoma and paraganglioma. Front Genet. (2022) 13:1022131. doi: 10.3389/fgene.2022.1022131 36568391 PMC9768187

[48] Theile D Haefeli WE Weiss J . Effects of adrenolytic mitotane on drug elimination pathways assessed in vitro. Endocrine. (2015) 49(3):842–853. doi: 10.1007/s12020-014-0517-2 25542188

[49] Boehm E Gill AJ Clifton-Bligh R Tothill RW . Recent progress in molecular classification of phaeochromocytoma and paraganglioma. Best Pract Res Clin Endocrinol Metab. (2024) 38(6):101939. doi: 10.1016/j.beem.2024.101939 39271378

[50] Qin S Xu Y Yu S Han W Fan S Ai W . Molecular classification and tumor microenvironment characteristics in pheochromocytomas. Elife. (2024) 12. doi: 10.7554/elife.87586.3 PMC10942623 38407266

[51] Batchu S Hakim A Henry OS Madzo J Atabek U Spitz FR . Transcriptome-guided resolution of tumor microenvironment interactions in pheochromocytoma and paraganglioma subtypes. J Endocrinol Invest. (2022) 45(5):989–998. doi: 10.1007/s40618-021-01729-8 35088383

[52] Martinelli S Amore F Canu L Maggi M Rapizzi E . Tumour microenvironment in pheochromocytoma and paraganglioma. Front Endocrinol. (2023) 14:1137456. doi: 10.3389/fendo.2023.1137456 37033265 PMC10073672

[53] Li L Liu S Guo Z Tang Y Zhang Y Qiu L . Molecular signatures of cancer stemness characterize the correlations with prognosis and immune landscape and predict risk stratification in pheochromocytomas and paragangliomas. Bioengineering. (2025) 12(3). doi: 10.3390/bioengineering12030219 40150683 PMC11939611

[54] Ma X Ji P Zheng J Cai S Xu K Sun Y . Neuro-tumor interactions in peripheral tumors. Cancer Metastasis Rev. (2026) 45(2). doi: 10.1007/s10555-026-10321-6 41844958

[55] Farhat NA Powers JF Shepard-Barry A Dahia P Pacak K Tischler AS . A previously unrecognized monocytic component of pheochromocytoma and paraganglioma. Endocr Pathol. (2019) 30(2):90–95. doi: 10.1007/s12022-019-9575-6 31001800 PMC6816458

[56] Uchihara M Tanabe A Kojima Y Shimoi T Maeshima AM Umamoto K . Immunohistochemical profiling of SSTR2 and HIF-2alpha with the tumor microenvironment in pheochromocytoma and paraganglioma. Cancers. (2024) 16(12). doi: 10.3390/cancers16122191 38927897 PMC11201597

[57] Hadrava Vanova K Uher O Meuter L Ghosal S Talvacchio S Patel M . PD-L1 expression and association with genetic background in pheochromocytoma and paraganglioma. Front Oncol. (2022) 12:1045517. doi: 10.3389/fonc.2022.1045517 36439433 PMC9691952

[58] Monteagudo M Calsina B Salazar-Hidalgo ME Martinez-Montes AM Pineiro-Yanez E Caleiras E . MAML3-fusions modulate vascular and immune tumour microenvironment and confer high metastatic risk in pheochromocytoma and paraganglioma. Best Pract Res Clin Endocrinol Metab. (2024) 38(6):101931. doi: 10.1016/j.beem.2024.101931 39218714

[59] Ehrhart-Bornstein M Hinson JP Bornstein SR Scherbaum WA Vinson GP . Intraadrenal interactions in the regulation of adrenocortical steroidogenesis. Endocr Rev. (1998) 19(2):101–143. doi: 10.1210/edrv.19.2.0326 9570034

[60] Bornstein SR Chrousos GP . Clinical review 104: Adrenocorticotropin (ACTH)- and non-ACTH-mediated regulation of the adrenal cortex: neural and immune inputs. J Clin Endocrinol Metab. (1999) 84(5):1729–1736. doi: 10.1210/jcem.84.5.5631 10323408

[61] Brand C Cherradi N Defaye G Chinn A Chambaz EM Feige JJ . Transforming growth factor beta1 decreases cholesterol supply to mitochondria via repression of steroidogenic acute regulatory protein expression. J Biol Chem. (1998) 273(11):6410–6416. doi: 10.1074/jbc.273.11.6410 9497372

[62] Le Roy C Maisnier-Patin K Leduque P Li JY Saez JM Langlois D . Overexpression of a dominant-negative type II TGFbeta receptor tagged with green fluorescent protein inhibits the effects of TGFbeta on cell growth and gene expression of mouse adrenal tumor cell line Y-1 and enhances cell tumorigenicity. Mol Cell Endocrinol. (1999) 158(1-2):87–98. doi: 10.1016/s0303-7207(99)00176-8 10630409

[63] Brand C Souchelnytskiy S Chambaz EM Feige JJ Bailly S . Smad3 is involved in the intracellular signaling pathways that mediate the inhibitory effects of transforming growth factor-beta on StAR expression. Biochem Biophys Res Commun. (1998) 253(3):780–785. doi: 10.1006/bbrc.1998.9829 9918804

[64] Nedaeinia R Najafgholian S Salehi R Goli M Ranjbar M Nickho H . The role of cancer-associated fibroblasts and exosomal miRNAs-mediated intercellular communication in the tumor microenvironment and the biology of carcinogenesis: a systematic review. Cell Death Discov. (2024) 10(1):380. doi: 10.1038/s41420-024-02146-5 39187523 PMC11347635

[65] Perge P Butz H Pezzani R Bancos I Nagy Z Paloczi K . Evaluation and diagnostic potential of circulating extracellular vesicle-associated microRNAs in adrenocortical tumors. Sci Rep. (2017) 7(1):5474. doi: 10.1038/s41598-017-05777-0 28710381 PMC5511159

[66] Le Tourneau C Hoimes C Zarwan C Wong DJ Bauer S Claus R . Avelumab in patients with previously treated metastatic adrenocortical carcinoma: phase 1b results from the JAVELIN solid tumor trial. J Immunother Cancer. (2018) 6(1):111. doi: 10.1186/s40425-018-0424-9 30348224 PMC6198369

[67] Ababneh O Ghazou A Alawajneh M Alhaj Mohammad S Bani-Hani A Alrabadi N . The efficacy and safety of immune checkpoint inhibitors in adrenocortical carcinoma: a systematic review and meta-analysis. Cancers. (2024) 16(5). doi: 10.3390/cancers16050900 38473262 PMC10931182

[68] Jimenez C Subbiah V Stephen B Ma J Milton D Xu M . Phase II clinical trial of pembrolizumab in patients with progressive metastatic pheochromocytomas and paragangliomas. Cancers. (2020) 12(8). doi: 10.3390/cancers12082307 32824391 PMC7465458

[69] Calsina B Pineiro-Yanez E Martinez-Montes AM Caleiras E Fernandez-Sanroman A Monteagudo M . Genomic and immune landscape of metastatic pheochromocytoma and paraganglioma. Nat Commun. (2023) 14(1):1122. doi: 10.1038/s41467-023-36769-6 36854674 PMC9975198

[70] Jimenez C Andreassen M Durand A Moog S Hendifar A Welin S . Belzutifan for advanced pheochromocytoma or paraganglioma. N Engl J Med. (2025) 393(20):2012–2022. doi: 10.1056/nejmoa2504964 41124218

[71] Cannarile MA Weisser M Jacob W Jegg AM Ries CH Ruttinger D . Colony-stimulating factor 1 receptor (CSF1R) inhibitors in cancer therapy. J Immunother Cancer. (2017) 5(1):53. doi: 10.1186/s40425-017-0257-y 28716061 PMC5514481

[72] Anderson AC Yanai I Yates LR Wang L Swarbrick A Sorger P . Spatial transcriptomics. Cancer Cell. (2022) 40(9):895–900. doi: 10.1016/j.ccell.2022.08.021 36099884

[73] Huang RH Wang LX He J Gao W . Application and prospects of single cell sequencing in tumors. Biomark Res. (2021) 9(1):88. doi: 10.1186/s40364-021-00336-2 34895349 PMC8665603

